# Temporal trends in pulmonary embolism prevalence in Greece during 2013–2017

**DOI:** 10.1186/s12889-021-10621-2

**Published:** 2021-03-21

**Authors:** Ioannis C. Lampropoulos, Dimitrios G. Raptis, Zoe Daniil, Sotirios K. Tasoulis, Vassilis P. Plagianakos, Foteini Malli, Konstantinos I. Gourgoulianis

**Affiliations:** 1 Respiratory Medicine Department, University of Thessaly, School of Medicine, Biopolis (Mezourlo), 41110 Larissa, Greece; 2 Department of Computer Science and Biomedical Informatics, University of Thessaly, Lamia, Greece; 3 Greek National Health Service Organization (EOPYY), Athens, Greece; 4 Respiratory Disorders Lab, Faculty of Nursing, University of Thessaly, Larissa, Greece

**Keywords:** Pulmonary embolism, Prevalence, Prescribing patterns, Epidemiology

## Abstract

**Background:**

Pulmonary embolism (PE) epidemiological data about the disease prevalence in the general population are unclear. The present study aims to investigate the prevalence of PE in Greece and the associated temporal trends for the years 2013–2017.

**Methods:**

Data on medical prescriptions for PE in the years 2013–2017 were provided by the Greek National Health Service Organization (EOPYY). Data on age, gender, specialty of the prescribing physician and prescription unit were provided as well.

**Results:**

The total number of medical prescriptions for PE for the study period was 101,426. Of the total prescriptions, 51% were issued by the Public Sector and 48% by the Private Sector. In 2013 the prevalence of PE was 5.43 cases per 100,000 citizens and increased constantly until 2017 with 23.79 cases per 100,000 population. Prevalence was higher in all years studied in the age group of 70–80 years. For the year 2017, we observed 69.35 cases per 100,000 population for subjects 70–80 years, followed by the ages 80–90 (60.58/100,000) and 60–70 years (56.47 /100,000). Females displayed higher PE prevalence than males and higher increasing trend.

**Conclusion:**

PE prevalence has an increasing trend throughout the years 2013–2017 while prevalence in females is higher than males and displays a higher increasing trend. Our results may be used to appropriately organize nationwide health care campaigns aiming at the diagnosis, treatment and prevention of PE.

**Supplementary Information:**

The online version contains supplementary material available at 10.1186/s12889-021-10621-2.

## Background

Venous thromboembolism (VTE) (i.e., pulmonary embolism (PE) and deep vein thrombosis) diagnosis and management remains difficult, mainly due to its’ multifactorial causes, that may include (but are not limited to) aging and/or cancer [1, 2]. The variation of annual incidence rates of VTE among countries is significant. For Greece, PE incidence ranges from 14 in 1998 to 30 per 100,000 persons for 2012 [3]. VTE incidence in the United States of America (USA) has increased significantly by 82% in recent years [4] which is possibly associated with the increasing use of Computed Tomography Pulmonary Angiography (CTPA) among other diagnostic tests [5]. In Sweden, PE diagnosis increased from 0.69/1000 to 0.76/1000 during 2011–2018 [6]. The characteristics of the different cohorts, like age and nationality, the retrospective retrieval of data from medical records, and the imperfect discrimination among primary and recurrent episodes [7] may be contributing factors for the differentiation among the studies.

It is remarkable that there are limited studies that aim to investigate trends in the incidence of PE. There is a remarkable variation around the world about the rates of PE incidence, due to both the differences in VTE risk factors and the differences in PE diagnosis among countries. In Australia and United Kingdom, PE related admissions have exhibited increasing trends [8, 9] although in other countries such as China, PE incidence seems to be stable while the mortality rate has decreased [10]. In USA, there is an increase in incidence although PE is associated with a significant fall in mortality [5, 11] while PE related mortality exhibits a declining trend in USA and China [5, 10, 11]. To our knowledge, epidemiological data on PE in Greece, are scarce and are limited to a reported increase in PE incidence for the period 1998 to 2012 [3].

PE diagnosis usually takes place in the hospital setting and more rarely a physician of the private sector diagnoses the disease and then (usually) refers the patient to a hospital facility for further evaluation. Outpatient physicians offer primary care services either at a private clinic that has signed a contract with the organization for social security fund, i.e. the National Organization for Healthcare Services Provision (EOPYY), or at the health centers of the National Primary Care Network (PEDY).

The aim of the present study was to investigate the temporal trends in the prevalence of PE in Greece during the years 2013–2017 by using existing health administrative data for a large number of outpatients. For the purpose of the study, we analyzed electronic prescriptions that were issued with a diagnosis of PE. The term “prescription” corresponds to the term “medical prescription” that is defined as a health care provider’s written authorization that is given to a patient in order to purchase a prescription drug from a pharmacy. The electronic prescription is entered into an electronic medical record system and then printed before being handed-out to a pharmacist.

## Methods

### Participants

The present study is a retrospective observational epidemiological study undertaken in a large cohort of Greek patients with a diagnosis PE. In more detail, we used electronic prescriptions that were issued for the ICD-10 codes I26.0 and I26.9 that correspond to Pulmonary embolism with and Pulmonary embolism without Acute Cor Pulmonale (i.e., acute right heart failure),   respectively.

### Data acquisition

Health administrative data were collected from the Center of e-prescription Data Processing (KMES) of the Greek National Health Service Organization (EOPYY). KMES is an electronic business intelligence system which analyzes the data from all the prescriptions issued (electronic and hand written) [12]. KMES platform records, fills, processes and analyzes all prescriptions that are recorded in the system and are executed by all the pharmacies. In Greece the e-prescribing penetration is more than 95% for outpatients suggesting that more than 95% of the medical prescriptions are issued electronically and are not hand written. EOPYY is the largest social security fund (SSF) that covers the majority (> 90%) of the population that has insurance. EOPPY was established in 2012 when the individual SSFs were merged and provides health insurance through contracted physicians, or health centers of PEDY.

In the present study, the KMES data provided to us relate to the period between January 2012 and December 2017. The year 2012 was a milestone year for EOPYY with simultaneous use of handwritten and electronic prescriptions. Therefore, the prescriptions issued in 2012 that were recorder in the electronic system with a diagnosis of PE were only 82 and did not actually correspond to the reality, so the data were discarded. From the year 2013, only electronic prescriptions were issued so the period under consideration sets out from January 2013 up to December 2017. The data that were provided to us by the KMES database have the following features: diagnosis of PE with a disease description (ICD_10 code of I26.0 for PE with Acute Cor Pulmonale and I26.9 for PE without Acute Cor Pulmonale), the date the prescriptions were issued and the date that they were carried-out by the pharmacist, pharmacy prefix, specialty of the prescribing physician (e.g. pulmonologist, cardiologist, hematologist), the prescription unit (e.g. Private Sector, Public Sector, Tertiary Sector, Primary care facility), and the number of prescriptions by Social Security Number (AMKA). The total number of electronic prescriptions provided to us was 101,426. The data correspond to individual Social Security Number so that we would not have patient replicates. We have assessed the prevalence of PE with and without Acute Cor pulmonale by using the different ICD10 codes in order to describe PE prevalence in more detail and to provide data on the prevalence of PE according to its severity.

The authorization to use the anonymized data was obtained by EOPYY [9th of January 2019 (09/01/2019) data acquisition and data access agreement for research purposes, presidents’ approval decision], in accordance with the applicable legislation on the protection and processing of personal data. Data on all patients’ identifiers were not provided to the authors in order to assure patient confidentiality. The study protocol was approved by the ethics committee of our institution.

### Statistical analysis

Demographic characteristics are reported as mean ± standard deviation, unless otherwise indicated. Datasets were tested for normality using the Shapiro-Wilk normality test. Incidence rates comparison was performed using a parametric t-test. All the statistical analysis was performed at the statistical significance level of 5% corresponding to a *p* value of 0.05.

The time period investigated concerns monthly observations for approximately 5 years (January 2013 to December 2017). We expected a positive correlation however we were not sure how strong it was and whether it behaved differently with respect to the categorical variable that separates samples in males and females. For this reason we inspected differences in intercept and slope for the corresponding regression lines. To achieve this, we employed the analysis of covariance (ANCOVA) in order to compare two or more regression lines. As previously reported [13], ANCOVA tests the effect of a categorical variable on a dependent factor (in this case prevalence) while controlling for the effect of a continuous co-factor (in this case time). As we are interested in comparing two regression lines, the categorical factor (in this case sex) splits the relationship between the two variables into two linear equations. In order to compare them we tested differences in slopes and intercepts. Differences in intercepts can be interpreted as differences in magnitude while difference in slopes is interpreted as differences in the rate of change. We initially tested slopes for the possible interaction among the covariate and the factor. If the interaction between the factor and the covariate does not differ significantly from zero, then we can assume that the slopes investigated are similar. When that was the case, we proceeded to examine differences in intercept values among regression lines.

Data were analyzed using SPSS software, version 22 (Statistical Package for Social Sciences Inc., 2003, Chicago, USA).

## Results

**Fig. 1:**
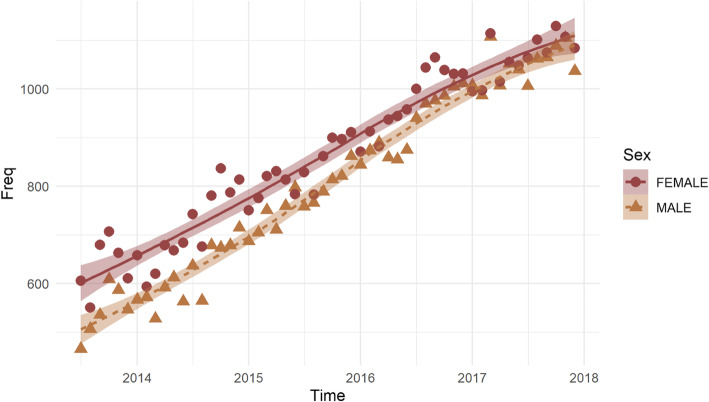
The two regression lines that correspond to male and female population respectively

The total number of e-prescriptions using a ICD10 code for PE, according to the EOPYY records, for the study period was 101,426. Table 1 presents the issuance of e-prescriptions in Greece. Of the total prescriptions, 51% (*n* = 51,654) were issued by the Public Sector, while 49,148 (48%) by the Private Sector. Further analysis revealed that the Primary Health Care Private Sector covered 45% (*n* = 45,633) of prescriptions, followed by the Secondary Health Care Public Sector with 24% (*n* = 24,610), the Primary Health Care Public Sector at 22% (*n* = 21,999) followed by the Tertiary Health Care Public Sector and the Secondary Health Care Private Sector (Fig. 1).

**Table 1:** The issuance of e-prescriptions in Greece

Parameter	e-prescriptions	%
Public sector	51,564	51%
Private Sector	49,148	49%
Primary Health Care, Private Sector	45,633	45%
Secondary Health Care, Public Sector	24,610	24%
Primary Health Care, Public Sector	21,999	22%
Tertiary Health Care, Public Care	4955	5%
Secondary Health Care, Public Sector	3515	3%
Primary Health Care, Public Benefit Organizations	671	1%
Without Information	43	0%

Τhe greater number of prescriptions was observed in the group of patients aged 70–80 years followed by the age group of 80–90 years and of 60–70 years (Table 2). The number of prescriptions in each age group was expressed per 100,000 age-matched population (Table 2).

**Table 2:** Absolute number of prescriptions sorted by age and number of prescriptions per 100,000 populations in each age group (prescriptions in age group/100,000 population of the same age group) for all study period

Age group (years)	Total prescriptions	Prescriptions per 100.000 population
0–10	1	0.10
10–20	308	28.71
20–30	1973	146.05
30–40	4555	278.54
40–50	8879	561.57
50–60	13,845	994.72
60–70	20,300	1790.05
70–80	25,091	2466.57
80–90	23,007	5419.14
90–100	3467	2401.70

We estimated the prevalence of PE and of PE with Acute Cor Pulmonale. The estimation of prevalence was based on the monthly e-prescriptions. Since e-prescriptions that are issued per patient are monthly, the average can equal the prevalence of PE for the study period. In 2013 the prevalence of PE without Acute Cor Pulmonale was estimated at 5.43 cases per 100,000 citizens and shows constant increase until 2017 with 23.79 cases per 100,000 population. In more detail, for females, in 2013 PE prevalence was estimated at 3.12 cases per 100,000 population and in 2017 at 12.76 cases per 100,000 while for males the prevalence increased from 2.30 cases per 100,000 population in 2013 to 11.03 cases in 2017 (Table 3). Additionally, we estimated prevalence per age group (Table S1). Prevalence is consistently higher in all years studied in the age group of 70–80 years with 69.35 cases per 100,000 population for the year 2017 followed by the ages 80–90 (60.58/100,000) and 60–70 (56.47/100,000) for the same year.

**Table 3:** Prevalence of PE in Greece for the study period for both genders. Prevalence corresponds to cases per 100,000 population

Years	Total prevalence	Male prevalence	Female prevalence
2013	5.43	2.3	3.12
2014	12.10	5.60	6.50
2015	16.22	7.26	8.97
2016	20.60	9.31	11.29
2017	23.79	11.03	12.76

As for PE with Acute Cor Pulmonale, its' prevalence presents an increase between 2013 and 2017, starting at 0.12 and reaching 0.18 per 100,000 population, respectively. Table 4 presents the prevalence of PE and of PE with Acute Cor Pulmonale for both sexes.

**Table 4:** Prevalence of PE with Acute Cor Pulmonale in the whole sample, in males and in females. Prevalence corresponds to cases per 100,000 population

Years	Prevalence of PE with Cor Pulmonale	Prevalence in males	Prevalence in females
2013	0.12	0.05	0.07
2014	0.17	0.08	0.10
2015	0.19	0.08	0.11
2016	0.19	0.08	0.11
2017	0.18	0.07	0.10

Abbreviations: *PE* Pulmonary embolism

We used regression to determine the causal relationship between the years studied, gender and PE prevalence. There is a significant effect of time and sex and also a significant interaction with PE prevalence. The slope of the regression between prevalence and time is not similar for males and females. Since slopes are significantly different between groups, then testing for different intercepts is not necessary. To this end, having tested the differences in regression lines we can fit linear regressions separately for males and females to investigate them further. Table S2 summarizes the results for both models (model 1 for males and model 2 for females, respectively). The regression lines indicate that females have a higher intercept and while regression slope is positive for both groups the prevalence grows faster for males. We may visually investigate the regression lines for males and females in Fig. 1 where observations with different color correspond to different groups.

The bulk of prescriptions are issued by pulmonologists with 3.16 prescriptions per physician, followed by hematologists with 1.99 prescriptions per physician. Tables 5 and 6 present in more detail prescriptions issued by specialization, the number of registered physicians in every specialization and the proportion of prescriptions both for PE without and for PE with Acute Cor Pulmonale issued by each specialty. We noticed that Pulmonologists issue most of the prescriptions both for patients with PE without and PE with Acute Cor Pulmonale. For PE with Acute Cor Pulmonale, cardiologists follow second, while in the case of PE without cor pulmonale Hematologists rank second.

**Table 5:** Prescriptions issued by specialist for patients diagnosed with PE without cor pulmonale

Pulmonary Embolism			
Specialty	ElectronicPrescriptions	Doctors per Specialty	Ratio of Prescriptions/ Doctors
Pulmonologistics	21,187	6708	3.16
Hematologists	4528	2277	1.99
General Medicine	18,766	12,990	1.44
Pathologists	25,792	19,404	1.33
Cardiologists	17,567	14,852	1.18
Onologists - Pathologists	918	1368	0.67
Vascularsurgeons	771	1274	0.61
Otherspecialties	11,897	202,704	0.06

**Table 6:** Prescriptions issued by specialist for patients diagnosed with PE with cor pulmonale

Pulmonary Embolism with reference to acute cor pulmonale			
Specialty	ElectronicPrescriptions	Doctors per Speciality	Ratio of Prescriptions/Doctors
Pulmonologists	264	6708	0.04
Cardiologists	240	15,852	0.02
Pathologists	284	19,704	0.01
General Medicine	181	12,990	0.01
Hematologists	31	2277	0.01
Otherspecialties	1000	57,531	0.02

Finally, we performed prevalence estimation by geographical region. Greece was divided into 4 geographical regions (that is, Attica – Northern Greece – Southern Greece – Islands). Table 7 presents prevalence by geographical region. The higher increase of prevalence is noticed in Attica with 5.95 cases per 100,000 population in 2013 while in 2017 the prevalence reached 29.47cases per 100,000. Next comes Northern Greece, then Southern Greece and last the Islands. For Attica and Northern Greece, the difference was highly significant with a *p* value< 0.01.

**Table 7:** Prevalence of PE in Greece per geographical region

Year	Attica	Northern Greece	Southern Greece	Ionian Islands
2013	5.95	5.72	4.09	1.88
2014	13.92	12.88	8.12	3.86
2015	20.05	15.94	11.71	4.75
2016	25.50	20.27	15.24	5.78
2017	29.49	23.29	16.98	7.07

## Discussion

The present study included data from 101,426 prescriptions for PE cases from Greece during 2013–2017. Our findings suggest that PE prevalence for Greece is 23.79 cases per 100,000 population for the year 2017 which is less than the prevalence reported by other researchers in foreign countries [4]. Moreover, we revealed that there is an ascending tendency in PE prevalence from 2013 to 2017 that may be due to advances in PE diagnostics. Age and gender distributions is in agreement with previous studies [3, 14].

The PE annual prevalence for 2017 was estimated to 23.79 per 100,000 population in Greece. The reported trends in PE are lower than those reported in the literature. To our knowledge, this is the first study to describe the prevalence of PE in the general population of a country. Most studies report data on PE prevalence for in-hospital population. The prevalence of PE has been reported as high as 37% among selected hospitalized patients [15] while others reported a prevalence of 0.6% for subjects reporting syncope in the emergency department [16] and 16.1% in patients suffering from acute exacerbation of Chronic Obstructive Pulmonary Disease [17]. We have estimated PE prevalence by assessing the number of prescriptions by Social Security Number so that we would not have patient replicates counted more than one time so our study gives a rather fair estimate of the disease prevalence on the general population of Greece.

We reported a significant annual increase in PE prevalence during the study period. PE prevalence raised from 5.43 cases per 100,000 population in 2013 to 23.79 in 2017. In the same context, a slight increase was observed in the prevalence of PE with acute right heart dysfunction. Our data revealed a female predominance of PE prevalence throughout the study period consistent with the higher frequency of the disease in females [3]. Our results are in accordance with earlier published data [3, 5, 8, 18–21], indicating a rising trend in PE case rates all over the years. We have previously reported an increase in PE incidence in Greece and a rather small mortality rate for the years preceding our study [3]. The increase in PE prevalence may be attributed to the wide availability and use of CT imaging among clinicians [5]. The correlation of the increase in PE prevalence may be additionally associated with increased prevalence of venous thromboembolism associated risk factors such as aging, heart failure and obesity [22, 23].

Our results provide further support to the age-dependent increase in VTE risk. We have observed increased PE prevalence in older subjects with a peak at the age groups of 70–80 years for both genders. Our findings are consistent with previously published data. Other studies showed that incidence rates of PE in elderly patients (≥65 years) are three times as high when compared to younger patients [14].

Additionally, in our research we found that the increase in PE prevalence was evident in both genders, although females have a higher rate of prevalence increase throughout the study period. The reasons underlying the gender differences cannot be addressed on the present study, however, we hypothesize that discrepancies in life expectancy amongst sexes may at least in part provide an explanation. During the study period, the life expectancy of females ranged from 84.00 to 83.90 years (from 2013 to 2017, respectively) while life expectancy in males varied from 78.70 to 78.80 (from 2013 to 2017, respectively) and constantly remained lower than those for females [24]. However, we acknowledge that PE prevalence is consistently higher in females than males in all age groups, therefore the discrepancies in life expectancy cannot be fully explained by the increased life expectancy in women. Additional factors, including differences in fibrinolytic and/or thrombogenic activity among genders may at least in part explain the sex-related discrepancies in PE prevalence [25]. Clearly, the present study was not designed to address these findings and further studies are warranted in order to assess the factors underlying the female predominance in PE prevalence.

In our study, we observed that patients with PE choose 51% of public health services versus 48% of the private sector. Also, we demonstrated that patients with PE are followed-up usually by a Pulmonologist than by other specialties. This probably reflects the distribution of PE hospitalizations in Greece where there is anecdotal evidence that patients suffering from PE (occurring in the outpatient setting) are hospitalized mainly in Respiratory Medicine Departments. Venous thromboembolism is a multifactorial disease and may require multidisciplinary approach involving almost any medical specialty but more commonly Pulmonologists, Cardiologists and Hematologists. Our results reflect the national distribution of PE follow-up trends and highlight the need for national training programs for PE that aim in these specialties (both in the Public and Private sector).

The present research has several strengths and limitations. To our knowledge there is no previous published data on PE prevalence in Greece. However, our study is of retrospective nature while we did not have available data on demographics of the cases (besides age, gender and health provider) or VTE related risk factors (like cancer or hormone-replacement therapy). Additionally, data on PE with acute cor pulmonale were based on physician reporting and not on a standard definition of acute cor pulmonale. Therefore, we suppose that most of the cases reported as PE with acute cor Pulmonale may reflect patients with High risk PE (according to international guidelines [26]), however we cannot exclude that some patients would be classified as Intermediate-high risk PE.

## Conclusion

In conclusion, this is the first report of PE prevalence in a nationwide general population of a country (Greece) where we report an increasing trend for PE prevalence throughout the years. Female prevalence is higher than males with a higher increasing trend. The present results may be used to effectively organize nationwide health care systems campaigns towards the diagnosis, treatment and prevention of PE.

## Supplementary Information

**Additional file 1: Table S1.** Pulmonary embolism prevalence per age group through the study period (2013-2017). Data are expressed per 100.000 population per each year. “Average” corresponds to the mean prevalence per age group throughout the whole study period. **Table S2.** Regression Results for Male and Female population separately.

## Data Availability

The data analyzed during the current study were available from the National Organization for Healthcare Services Provision (EOPYY) and are presented within the paper. The detailed clinical data are not publicly available in order to ensure study subjects anonymity and protect confidentiality. Data will be available upon request to the corresponding author with permission from EOPPY.

## Abbreviations

VTE: Venous Thromboembolism
PE: Pulmonary Embolism
CTPA: Computed Tomography Pulmonary Angiography
USA: United State of America
EOPYY: National Organization for Healthcare Services Provision
PEDY: National Primary Care Network
KMES: Center of e-prescription data processing
SSF: Social Security Fund
AMKA: Social Security Number
SPSS: Statistical Package for Social Sciences
CT: Computed Tomography
