# Supplementary material for: Temporal trends in pulmonary embolism prevalence in Greece during 2013–2017
Source: BMC Public Health. 2021 Mar 21;21:559. doi: 10.1186/s12889-021-10621-2 (PMC7981863; doi:10.1186/s12889-021-10621-2)

Table S1. Pulmonary embolism prevalence per age group through the study period (2013-2017). Data are expressed per 100.000 population per each year. “Average” corresponds to the mean prevalence per age group throughout the whole study period.

| Age group (years) | 0-10 | 10-20 | 20-30 | 30-40 | 40-50 | 50-60 | 60-70 | 70-80 | 80-90 | 90-100 |
| --- | --- | --- | --- | --- | --- | --- | --- | --- | --- | --- |
| Year |  |  |  |  |  |  |  |  |  |  |
| 2013 | 0.00 | 0.20 | 0.63 | 2.67 | 4.84 | 8.76 | 13.05 | 17.72 | 15.03 | 2.23 |
| 2014 | 0.00 | 0.55 | 2.07 | 5.46 | 10.87 | 20.50 | 29.29 | 36.45 | 34.91 | 5.11 |
| 2015 | 0.00 | 0.83 | 3.63 | 8.69 | 16.36 | 25.50 | 38.85 | 47.67 | 46.70 | 6.46 |
| 2016 | 0.00 | 0.61 | 5.93 | 11.26 | 21.97 | 32.98 | 50.03 | 60.78 | 55.49 | 8.15 |
| 2017 | 0.01 | 0.65 | 5.98 | 14.03 | 28.05 | 40.26 | 56.47 | 69.35 | 60.58 | 10.10 |
| **Average** | **0.00** | **0.57** | **3.65** | **8.42** | **16.42** | **25.60** | **37.54** | **46.39** | **42.54** | **6.41** |

*Table S2: Regression Results for Male and Female population separately.*


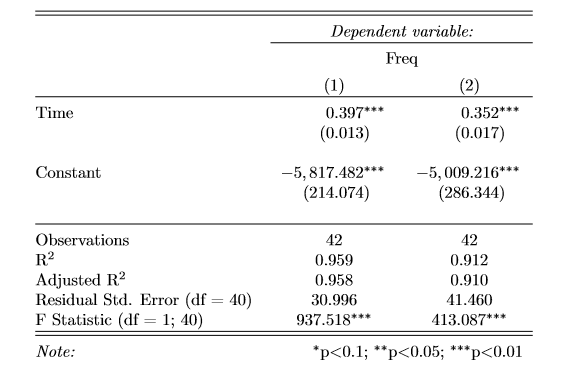

Supplement: Supplementary file 1 — Additional file 1: Table S1. Pulmonary embolism prevalence per age group through the study period (2013-2017). Data are expressed per 100.000 population per each year. “Average” corresponds to the mean prevalence per age group throughout the whole study period. Table S2. Regression Results for Male and Female population separately. [file 12889_2021_10621_MOESM1_ESM.docx]
